# Supplementary material for: The SCCmec Types and Antimicrobial Resistance among Methicillin-Resistant Staphylococcus Species Isolated from Dogs with Superficial Pyoderma
Source: Vet Sci. 2021 May 13;8(5):85. doi: 10.3390/vetsci8050085 (PMC8153549; doi:10.3390/vetsci8050085)
Supplement: Supplementary file 1 [file vetsci-08-00085-s001.zip › Table-S1-MPCR1.pdf]

Table S1. M-PCR1 for amplification of *ccr* gene complex type with *mecA* (Kondo et al., 2007)

Primer for PCR	Nucleotide sequence (5'-3')	Constructed on:	Reference SCCmec or SCC sequence(s)[a]	Gene(s) or gene allele(s) detected (primer pair)	Expected size of product (bp)
mA1	TGCTATCCACCCTCAAACAGG	*mecA*	Type II.1	*mecA* (mA1-mA2)	286
mA2	AACGTTGTAACCACCCCAAGA	*mecA*	Type II.1		
α1	AACCTATATCATCAATCAGTACGT	*ccrA1*	Type I.1	*ccrA1-ccrB* (α1-ßc)	695
α2	TAAAGGCATCAATGCACAAACACT	*ccrA2*	Type II.1	*ccrA2-ccrB* (α2-ßc)	937
α3	AGCTCAAAAGCAAGCAATAGAAT	*ccrA3*	Type III.1	*ccrA3-ccrB* (α3-ßc)	1,791
ßc	ATTGCCTTGATAATAGCCITCT	*ccrB1*, *ccrB2*, *ccrB3*	Type I.1, II.1, III.1		
α4.2	GTATCAATGCACCAGAACTT	*ccrA4*	Type VI	*ccrA4-ccrB4* (α4.2-ß4.2)	1,287
ß4.2	TTGCGACTCTCTTGGCGTTT	*ccrB4*	Type VI		
ɣR	CCTTTATAGACTGGATTATTCAAAATAT	*ccrC*	SCC*mercury*, Type V	*ccrC* (ɣR-ɣF)	518
ɣF	CGTCTATTACAAGATGTTAAGGATAAT	*ccrC*	SCC*mercury*, Type V		

[a] Accession numbers deposited in DDBJ/EMBL/GenBank database used as reference sequences for *SCCmec* elements and *SCCmercury* are as follows: type I.1 *SCCmec*, AB033763; type II.1 *SCCmec*, D86934; type II.2 *SCCmec*, AB127982; type II.3 (type IIE) *SCCmec*, AJ810120; type II.4 *SCCmec*, AB261975; type III.1 *SCCmec* and *SCCmercury*, AB037671; type IV.1 *SCCmec*, AB063172; type IV.*2 SCCmec*, AB063173; type IV.3*SCCmec*, AB096217; type IV.4 *SCCmec*, AB097677; type V *SCCmec*, AB121219; type VI *SCCmec*, AF411935.
