# Supplementary material for: The SCCmec Types and Antimicrobial Resistance among Methicillin-Resistant Staphylococcus Species Isolated from Dogs with Superficial Pyoderma
Source: Vet Sci. 2021 May 13;8(5):85. doi: 10.3390/vetsci8050085 (PMC8153549; doi:10.3390/vetsci8050085)
Supplement: Supplementary file 1 [file vetsci-08-00085-s001.zip › Table-S2-MPCR2.pdf]

Table S2. M-PCR2 for amplification of *mec* gene complex class (Kondo et al., 2007)

Primer for PCR	Nucleotide sequence (5'-3')	Constructed on:	Reference SCCmec or SCC sequence(s)[a]	Gene(s) or gene allele(s) detected (primer pair)	Expected size of product (bp)
mI6	CATAACTTCCCATTCTGCAGATG	*mecI*	Type II.1	*mecA-mecI* (mA7-mI6)	1,963
IS7	ATGCTTAATGATAGCATCCGAATG	IS*1272*	Type I.1	*mecA*-IS*1272* upstream of *mecA* (mA7-IS7)	2,827
IS2(iS-2)	TGAGGTTATTCAGATATTTCGATGT	IS*431*	Type V	*mecA*-IS*431* upstream of *mecA* (mA7-IS2 iS-2)	804
mA7	ATATACCAAACCCGACAACTACA	*mecA*	Type I.1, II.1, V		

[a] Accession numbers deposited in DDBJ/EMBL/GenBank database used as reference sequences for *SCCmec* elements and *SCCmercury* are as follows: type I.1 *SCCmec*, AB033763; type II.1 *SCCmec*, D86934; type II.2 *SCCmec*, AB127982; type II.3 (type IIE) *SCCmec*, AJ810120; type II.4 *SCCmec*, AB261975; type III.1 *SCCmec* and *SCCmercury*, AB037671; type IV.1 *SCCmec*, AB063172; type IV.*2 SCCmec*, AB063173; type IV.3*SCCmec*, AB096217; type IV.4 *SCCmec*, AB097677; type V *SCCmec*, AB121219; type VI *SCCmec*, AF411935.
